# Fostering Strategies to Expand the Consumption of Edible Insects: The Value of a Tripartite Coalition between Academia, Industry, and Government

**DOI:** 10.1093/cdn/nzy056

**Published:** 2018-06-15

**Authors:** Joel B Mason, Richard Black, Sarah L Booth, Andrew Brentano, Bill Broadbent, Peggy Connolly, John Finley, Jarrod Goldin, Tim Griffin, Kelly Hagen, Julie Lesnik, Gabi Lewis, Zhongli Pan, Juan Morales Ramos, Mark Ranalli, Guadalupe Rojas, Marianne Shockley, Valerie J Stull, Dariusz Swietlik

**Affiliations:** 1 USDA Human Nutrition Research Center at Tufts University, Boston, MA; 2 Friedman School of Nutritional Science and Policy, Tufts University, Boston, MA; 3 Quadrant D Consulting, West Harrison, NY; 4 Tiny Farms, Inc., San Leandro, CA; 5 Entomarket.com, Auburn, ME; 6 USDA–Agricultural Research Service, Beltsville, MD; 7 Entomo Farms, Norwood, Ontario, Canada; 8 Department of Anthropology, Wayne State University, Detroit, MI; 9 EXO, Inc., Brooklyn, NY; 10 USDA Western Regional Research Center, Albany, CA; 11 National Biological Control Laboratory, Leland, MS; 12 Tufts University School of Engineering and Gordon Institute, Medford, MA; 13 Department of Entomology, University of Georgia, Athens, GA; 14 Center for Sustainability and the Global Environment, University of Wisconsin–Madison, Madison, WI

**Keywords:** edible insects, coalition, food sustainability, cricket, whitepaper

## Abstract

Although many insect-based foods are nutritious and often an inexpensive option for human and domesticated animal consumption, there remains a negligible market for such foods in many countries. Several environmental and economic considerations underscore the potential value of insect-based foods, and emerging science suggests that diets incorporating such foods might also convey some genuine health benefits. However, if expanded markets for insect-based foods in cultures naïve to entomophagy are to be pursued, it will be important to develop multifaceted and coordinated strategies to *1*) delineate authentic health benefits, *2*) explore means of optimizing insect husbandry and food processing, *3*) examine cultural barriers to acceptance, *4*) formulate workable approaches to marketing, and *5*) address relevant food regulations. We sought to construct a multidisciplinary coalition whose goals are to investigate the above-mentioned 5 issues. Eighteen individuals from government, industry, and academia, with collective expertise in the fields of entomology, insect husbandry, human nutrition, sustainable agriculture, entomophagy, consumer product development and marketing, food-processing technologies, food regulatory affairs, and the anthropology of food selection, convened a 1-d summit and formed a tripartite organization to integrate their varied perspectives. Collaborative efforts are underway among members of this coalition to accomplish these multiple goals. Coordinating efforts between accomplished experts in relevant fields of academia, government, and industry will greatly expand our knowledge of and appreciation for the potential benefits of insect-based foodstuffs to individuals, to society, and to the sustainability of the global food supply, and thereby inform us as to how to proceed in a judicious and intelligent manner.

“Primates need good nutrition…not only fruits and plants, but insects as well.”

—Richard Leakey, Paleoanthropologist; Professor, Stony Brook University and Chair of the Turkana Basin Institute

Insect-based foodstuffs have been an integral component of the diet of many cultures for centuries (1, 2), having established themselves as nutritious and inexpensive foods. However, there remains a negligible market for the sale and consumption of such foods in several regions, including North America and Europe. Furthermore, even in those countries where entomophagy is firmly established, there is considerable opportunity to improve the productivity and healthfulness of insect husbandry and processing. Similarly, insect-based feed for domestic animals represents an underutilized source of high-quality protein and calories (3, 4).

Despite its relative absence in the food stream of many countries, the regular inclusion of insect-derived foodstuffs into the diets of humans and livestock offers a number of compelling environmental and economic benefits (5–8), as outlined below. Therefore, a thoughtful examination of whether such changes in the diet are warranted—and how they would be best accomplished—is in order. Moreover, although research on the topic is in its infancy, there is emerging evidence that substituting insect-based foodstuffs for more conventional foods sourced from domestic mammals may offer genuine health benefits (9–13). Thus, increasing the consumption of insect-based foodstuffs not only will convey tangible benefits to society and the environment but offers a real potential for promoting the health of individuals. Furthermore, the reasons to promote this shift in dietary habits are becoming increasingly relevant and urgent as the world population burgeons toward 9 billion individuals and the issues of limited natural resources and food sustainability become ever more pressing [e.g., (14, 15)]. The major purpose of this article, nevertheless, is not to elaborate on the environmental, economic, and health benefits of insect-based foodstuffs because this was done quite elegantly—and in exhaustive detail—in a 2013 treatise published by the UN FAO, entitled “Edible insects: future prospects for food and feed security” (5). Rather, our purpose in this article is to announce the establishment, and explain the rationale and underlying principles, of a working group whose mission is to explore the diverse array of potential benefits accompanying the increased consumption of insect-based foods and to define the strategies that would be needed to establish a robust market for these foods. The name of this group is TOPIC (Tripartite Organization for the Promotion of Insect Consumption).

However, a brief synopsis of the environmental benefits to be gained is worthy of mention. As the world's population continues to increase, the amount of arable land is decreasing due to erosion, climate-related conditions, and demographic trends. We are in a race to increase crop production and find new food sources that are less destructive of our finite natural resources of land and water, and less impactful on the atmosphere (6). Including edible insects as a significant source of human nutrition and animal feed can relieve much of this destructive pressure because the conversion ratio of feed to nutritious biomass is 2- to 12-fold greater for insects compared with poultry, fish, and domestic farm mammals (5, 8). The cultivation of insects may be a sustainable option for many reasons including the following:

- Higher food-conversion efficiency (cricket flour is twice as high as chicken and pork, 4 times that of sheep, and 6 times that of cattle) (5, 8)
- Higher percentage of consumable and digestible mass (mealworms, 100%; crickets, 80%; chicken, 55%; and beef, 40%) (5)
- Lower water requirements per gram of protein produced (cricket = 0.7–0.8 g, chicken = 5.2 g, cattle = 16.8 g) (5, 7)
- Lower greenhouse gas emissions in grams per kilogram of biomass produced (methane: crickets = 0.1, pigs = 1.9, cattle = 114; carbon dioxide: crickets = 7.6, pigs = 79.6, cattle = 285) (5, 8)

## TOPIC: A Coalition with Unique Strengths and Capabilities

Although the members of TOPIC come from highly diverse backgrounds, the common goal shared by this newly formed North American triad of experts from academia, industry, and government is to explore the wide diversity of potential benefits resulting from the regular consumption of insect-based foods, as well as potential obstacles that could hinder the creation of an expanded market. More specifically, the initial aims of TOPIC will be as follows: *1*) delineate authentic health benefits and articulate risks, *2*) explore means of optimizing insect husbandry and food processing, *3*) examine cultural barriers to acceptance, *4*) formulate workable approaches to marketing, and *5*) address relevant food regulations. By doing so, intelligent, informed, and rational choices can be made as to whether, and how, these foods should assume a larger role as an acceptable, economical, and sustainable component of the global food supply. Thus, the strength of the collaboration is the common goal of examining insects as a nutritious food for humans and domesticated animals. Participants to date include those with expertise in the fields of entomology and insect husbandry, human nutrition, sustainable agriculture, entomophagy, consumer product development and marketing, food-processing technologies, food regulatory affairs, and the anthropology of food selection.

Until now, scientists, policymakers, and commercial interests have pursued this path on very separate tracks, and often at odds with one another. However, although the short-term goals of these 3 contingents differ in some respects, the long-term objective—developing strategies to expand the consumption of insect-based foods—is the same. At a summit convened in April 2017 by the Jean Mayer USDA Human Nutrition Research Center on Aging at Tufts University in Boston, 17 people, representing industry (*n* = 6), government (*n* = 6), and academia (*n* = 5), came together for a 1-d meeting and agreed to form this coalition (one Agricultural Research Services official is an inaugural member of TOPIC but could not attend the summit meeting) (**Table 1**). As plans continue to evolve, we expect that this core group will expand to include other key leaders in each of these 3 sectors.

**TABLE 1:** Founding members of TOPIC^1^

Name, affiliation	Field of expertise
Industry	
Richard Black, Quadrant D Consulting	Food product marketing
Andrew Brentano, Tiny Farms, Inc.	Commercial insect husbandry, processing
Bill Broadbent, EntoMarket.com	Commercial insect husbandry, processing
Jarrod Goldin, Entomo Farms	Commercial insect husbandry, food processing
Kelly Hagen, Entomo Farms	Commercial insect husbandry, food processing
Gabi Lewis, EXO, Inc.	Commercial cricket foodstuffs
Academia	
Sarah Booth, Jean Mayer USDA Human Nutrition Research Center at Tufts University	Human nutrition science, micronutrients
Tim Griffin, Tufts University Friedman School of Nutrition Science and Policy	Domestic and global food security, agricultural methods
Julie Lesnik, Wayne State University	Anthropology of food selection
Joel Mason, USDA Human Nutrition Research Center at Tufts University	Human nutrition science, nutrition, and cancer prevention
Mark Ranalli, Tufts University Gordon Institute	Development of entrepreneurial enterprises
Marianne Shockley, University of Georgia	Community outreach programs in entomophagy
Valerie Stull, University of Wisconsin	Postdoctoral fellow examining health benefits of cricket consumption
Government	
John Finley, USDA-ARS	Government perspectives on food sustainability, regulatory affairs
Zhongli Pan, USDA-ARS	Food-processing technologies
Juan Morales Ramos, USDA-ARS	Insect husbandry
Guadalupe Rojas, USDA-ARS	Insect husbandry
Dariusz Swietlik, USDA-ARS^2^	Government perspectives on food sustainability, regulatory affairs

1 ARS, Agricultural Research Service; TOPIC, Tripartite Organization for the Promotion of Insect Consumption.

2 Founding member, but could not attend the inaugural meeting.

Although persons around the world consume a wide variety of insects, the initial efforts of the group will focus on crickets and cricket powder. The latter consists of roasted, whole crickets ground to a powder and is commonly referred to as “cricket flour” because it can serve as a flour substitute in many prepared foods, although its protein content is ∼5-fold greater than whole-wheat flour and its constituents are unique in other ways as well. This initial focus was chosen because *1*) crickets are hardy animals and the technology of cricket husbandry has evolved considerably in the past decade, *2*) cricket products are already the most commonly produced insect-based foodstuff for North American and European markets, and *3*) cricket flour can be readily incorporated into many foods with little alteration in texture, taste, appearance, and palatability, therefore circumventing the initial aversion that insect-naïve consumers often have toward eating insects. North American entrepreneurs have already shown, on a small scale, the viability of using cricket-based products—primarily protein bars, snack chips, and as novel cuisine offerings in an increasing number of restaurants. This emerging interest is also reflected by a large increase in references to edible insects in the popular press and peer-reviewed publications. For example, 973 citations are listed under “edible insects” in Google Scholar from January 2017 through May of 2018 compared with 95 citations in the years 2000–2001 (16).

Coordinating efforts between academia, government, and industry carries with it tremendous advantages (17). For instance, the best efforts by industry to introduce novel foods are sometimes thwarted when governmental regulations based on outdated standards are not updated and developed in parallel. Shifts in governmental policy often prompt industry to reconfigure priorities and assume new perspectives. An example of progress on reducing governmental barriers recently occurred in Europe: updated regulations instituted in the European Union, which took effect on 1 January 2018, recognize the legitimacy of whole insect foods, thereby facilitating applications from insect food manufacturers seeking approval to market insect-based foods in the European Union (18). Scientists within academia have much to contribute as well because they offer various research capabilities by which novel methodologies can be developed to improve husbandry, processing, and food technology. Other academic scientists focus on the sociological aspects of foods, providing insights as to how to contend with cultural barriers to acceptance, and importantly, still other academics are busily exploring as-of-yet undiscovered health benefits [and unintended side effects (19, 20)] that might accompany insect consumption. Industry also possesses invaluable strengths that neatly complement the other 2 legs of this coalition: it possesses a level of sophistication in generating means of large-scale production, effective marketing, and distribution in a sustainably profitable manner that the other 2 sectors often lack. In combination, therefore, the individual spheres of expertise offered by these 3 sectors provide the knowledge and necessary perspectives required to create an integrated blueprint to study the nutritional and health value of insect-based foods, develop cost-efficient means of producing desirable and healthful food products, and construct effective strategies to address potential obstacles such as limitations in consumer acceptance. This cross-disciplinary group was convened with the support of the USDA’s research arm, the ARS, and its National Program Leader for Nutrition, which is encouraging a systems approach “to addressing the interacting elements of agricultural production including genetics, environment, management and post-harvest/socioeconomic factors as a whole, and not just as a collection of parts.” The level of enthusiasm was at a high pitch because all participants sensed the considerable opportunities for synergy created by integrating these 3 sectors. The coalition recognizes that efforts to examine the value of insect-based foods are occurring in many countries outside of North America, and therefore, as the coalition evolves, soliciting advice and partnership with foreign groups possessing more experience will be valuable.

## Initial Priorities

Discussions at the inaugural TOPIC meeting identified 3 overarching categories of objectives that coalition members judged to be important in order to successfully create large and financially viable markets of healthful insect-based foodstuffs. The 3 are as follows: *1*) optimizing production, *2*) exploring health implications, and *3*) market development. Major issues within each category, outlined below, will be prioritized so that initial efforts are channeled in an efficient manner.

### 1. Optimizing production

- How can methods of raising crickets be improved to reduce costs, standardize the product, and increase production to levels required for wide distribution?
- Similarly, how can food-processing technologies be improved to reduce costs, standardize the product, and increase production to levels required for wide distribution?
- What rearing and processing methodologies are needed to optimize nutritional content? Related to this is the need to identify the content of those nutrients that are most important to optimize.
- Insects can be reared on numerous foods that would otherwise be discarded. This offers the opportunity to repurpose agricultural and food wastes. What waste streams would best be utilized to improve food sustainability and address environmental concerns and what governmental regulations might need to be addressed in order for this to occur?

### 2. Health impacts

- The profile of macronutrients contained in insects differs substantially from that found in poultry, fish, mammals, and plant-derived foods; do these macronutrient profiles convey health benefits compared with the more conventional food sources?
- Does the profile of micronutrients offer any health benefits? For example, the iron content of whole crickets is ∼3-fold greater than beef (10) and the vitamin B-12 content of cricket flour is 10-fold greater than that contained in beef (Maxxam Analytics, Mississauga, Canada; 2017).
- Given the unique profile of macro- and micronutrients in crickets, are there any detriments to health that accompany regular consumption?
- Do chitin and chitosan, the primary components of the insect exoskeleton, have nutritional and physiologic benefits? Harms?
- Do edible crickets and cricket-based foodstuffs possess any antinutrient properties or allergens or exhibit mineral binding or bioaccumulation of toxins?

### 3. Market development

- What strategies can effectively address the esthetic aversion to insect consumption possessed by societies that are not accustomed to entomophagy?
- What markets provide ideal entry points into expanding insect consumption, and what marketing approaches succeed?
- What food-processing technologies need to be developed to improve the stability and palatability of cricket-based foodstuffs?
- What other insects or insect products should be developed for human and domestic animal consumption?

## Future Directions

As indicated above, TOPIC has set forth for itself an ambitious agenda of initial priorities. However ambitious these initial directions might be, there remain many other important issues that were not selected as initial foci of emphasis, but which are subjects that the coalition hopes to address in the future. For example, the issue of using insects, and their by-products, as a source of food for farm animals, house pets, and fertilizer is not included as an initial priority. Although the activities of this coalition are in their early stages, however, it has already become quite evident that the benefits obtained by integrating perspectives from academia, industry, and government are of great value and will assist in whatever issues the coalition chooses to tackle in the future.

## Notes

Supported in part by the Agricultural Research Service cooperative agreement 58-1950-0-014 (to JBM).

Author disclosures: JBM, RB, SLB, PC, JF, TG, JL, ZP, JMR, MR, GR, MS, VJS, and DS, no conflicts of interest. AB, BB, JG, KH, and GL are employees and/or owners of their respective insect food companies listed above.
